# Extrachromosomal circular DNA as a vehicle to gene transfer in plants

**DOI:** 10.1093/plphys/kiad380

**Published:** 2023-07-03

**Authors:** Lara Pereira, Luke T Dunning

**Affiliations:** Assistant Features Editor, Plant Physiology, American Society of Plant Biologists, Rockville, MD, USA; Ecology and Evolutionary Biology, School of Biosciences, University of Sheffield, Sheffield S10 2TN, UK; Ecology and Evolutionary Biology, School of Biosciences, University of Sheffield, Sheffield S10 2TN, UK

Adaptation to environmental change is necessary in all organisms, especially in sessile organisms such as plants. Increased gene expression is one of the core stress responses, but there can be limits to how much can be transcribed from a single locus. The obvious solution to this problem is to duplicate the needed genes, but how can this be achieved within a generation? Gene amplification through extrachromosomal circular DNA (eccDNA) is one form of genome plasticity capable of driving this process (Peng et al. 2022). These circular molecules have been observed in all kinds of eukaryote cells, where they can vary in size and genetic content. In plants, most eccDNA contain repetitive sequences, intergenic regions, and genic fragments, but it is rare to find full endogenous protein-coding genes. A well-known exception is a 400-kb eccDNA initially found in the crop weed *Amaranthus palmeri*, which carries 59 protein-coding genes, including the enzyme 5-enolpyruvylshikimate-3-phosphate synthase (EPSPS), target of the herbicide glyphosate (Koo et al. 2018). Remarkably, the increased gene copy number of *EPSPS* on the eccDNA molecules confers herbicide resistance.

The sequence, structure, copy number, and form of inheritance of the *EPSPS* replicon were previously investigated through fluorescence in situ hybridization (FISH) (Koo et al. 2018). The *EPSPS*-FISH signals showed that this important herbicide resistance gene was not integrated in the genome but presented as extrachromosomal elements, yet they anchored to the chromosomes during metaphase. In somatic cells, there were up to 80 copies of the replicon, and they were able to be transmitted to the progeny via chromosome tethering. Crossing sensitive and resistant *A. palmeri* plants showed that the replicon was inherited by most offspring, but the *EPSPS* copy number varied drastically among F_1_ individuals, tissues, and even cells.

Once sexual transmission of the eccDNA within *A. palmeri* was demonstrated, an interesting question was whether the replicon could also be passed to other species. Plants resistant to glyphosate were found for *Amaranthus spinosus*, another weed from the same genus (Nandula et al. 2014). In resistant *A. spinosus*, the *EPSPS* gene was overexpressed compared with susceptible individuals, and the genetic sequence of the *EPSPS* gene was identical to the copy in the glyphosate-resistant *A. palmeri* plants. Thus, the authors hypothesized that the eccDNA replicon driving herbicide resistance in both species was transferred during interspecific hybridization.

In a recent issue of *Plant Physiology*, Koo et al. (2023) demonstrated that the eccDNA replicon conferring glyphosate resistance in *Amaranthus* species can indeed be passed among species via both natural and experimental hybridization. FISH experiments showed that the eccDNA is present in resistant *A. spinosus*, as well as in F_1_ hybrids derived from crosses between resistant *A. palmeri* and susceptible *Amaranthus tuberculatus*. The authors also showed that the chromosome tethering, and therefore eccDNA distribution after cell division, is random, generating vast somatic mosaicisms for copy number variation.

This Research Report establishes that eccDNA can be passed among species via pollen, contributing to rapid adaptation when the replicon contains a favorable gene, as in the case of glyphosate resistance. However, it can be inferred that many other eccDNA are similarly transferred within and between species in nature, but because no relevant phenotype is associated, they are not tracked and studied.

In yeast, genetic material contained in eccDNA was shown to be reinserted in the chromosome locus after gene amplification (Demeke et al. 2015). Whether genomic fragments contained in eccDNA can be later integrated in the genome in plants is a question that remains to be resolved. Because they are to a major extent composed of repetitive sequences, including transposable elements (Lanciano et al. 2017), and linear and circular structures co-exist within the cell (Koo et al. 2018, 2023), it is likely that random integrations do occur as seen in yeast. These, however, would be potentially lost in the following generations unless they confer an evolutionary advantage.

Although in the Research Report these advances are discussed in the context of hybridization, the mechanism showed here could pave the way to further investigate the dynamics of lateral gene transfer in plants. In a recent review, we presented and discussed the most likely mechanisms contributing to grass-to-grass lateral gene transfer (Pereira et al. 2022). We concluded that reproductive contamination—defined as incorporation of alien genetic material as a result of illegitimate pollination—was the most likely mechanism to allow exogenous DNA within the host cell and that transposable elements and eccDNA were probably the vehicles for DNA integration. Here, Koo et al. (2023) illustrated that eccDNA is indeed a vehicle to pass genetic material among species via pollen, a groundbreaking finding that challenges Mendelian genetics and that could blur the boundaries of sexual reproduction.
